# Biological performance of a bioabsorbable Poly (L-Lactic Acid) produced in polymerization unit: *in vivo* studies

**DOI:** 10.12688/f1000research.73754.1

**Published:** 2021-12-13

**Authors:** Mariana Xavier, Nayla Farez, Paola Luciana Salvatierra, Andre Luiz Jardini, Paulo Kharmandayan, Sara Feldman

**Affiliations:** 1 School of Chemical Engineering, Institute of Biofabrication, Campinas, São Paulo, 13081-970, Brazil; 2 School of Medicine, LABOATEM Laboratory of Molecular Biology, Tissue Engineering and Emerging Therapies, Rosario, Santa fé, 2000, Argentina; 3 School of Medical Sciences, Campinas, São Paulo, 13083-970, Brazil; 4 Institute of Basic and Applied Sciences, Villa Maria, Cordoba, X5900, Argentina

**Keywords:** biomaterials, material synthesis, PLLA, polymers, orthopedic, tissue-engineering, biofabrication

## Abstract

**Background: **The biomaterials engineering goal is to manufacture a biocompatible scaffold that adequately supports or improves tissue regeneration after implantation of the biomaterial in the injured area. Many requirements are demanded for a biomaterial, such as biocompatibility, elasticity, degradation time, and a very important factor is its cost of importation or synthesis, making its application inaccessible to some countries. Studies about biomaterials market show that Polylactic acid (PLLA) is one of the most used polymers, but expensive to produce. It becomes important to prove the biocompatibility of the new PLLA and to find strategies to produce biocompatible biopolymers at an acceptable production cost.

**Methods**: In this work, the polylactic acid biomaterial was synthesized by ring-opening polymerization. The polymer was submitted to initial *in vivo* biocompatibility studies in 12 New Zealand female rabbits, assigned to two groups: (1) Lesion and PLLA group (n = 6), (2) Lesion No PLLA group (n = 6). Each group was divided into two subgroups at six and nine months post-surgical time. Before euthanasia clinical and biochemical studies were performed and after that tomographic (CT), histological (Hematoxylin and Eosin and Masson's trichrome) and histomorphometric analyses were performed to evaluate the injury site and prove biocompatibility. The final cost of this polymer was analyzed.

**Results: **The statistical studies of hemogram and hepatocyte enzymes, showed that there were no significant differences between the groups for any of the times studied, in any of the variables considered and the results of CT and histology showed that there was an important process of neoregeneration. The cost analysis showed the biopolymer synthesis is between R$3,06 - R$5,49 cheaper than the import cost.

**Conclusions:** It was possible to synthesize the PLLA biopolymer by cyclic ring opening, which proved to be biocompatible, potential osteoregenerative and cheaper than other imported biopolymers.

## Introduction

Tissue regeneration has currently attracted significant attention because it offers potential solutions for the treatment of many diseases. To produce scaffolds and other structures, it is essential to understand biomaterials because they must mimic the body's natural structures ^1^ The use of these materials is significant in cardiology, orthopedics, plastic surgery, wound healing and other areas. Bone substitutes are being increasingly used especially in oncologic surgery, traumatology, revision prosthetic surgery, and spine surgery ^2^. Bone grafting frequency is indeed the second most frequent tissue transplantation worldwide, with more than 500,000 implanted in the US alone ^3^.

A wide variety of bone substitutes have been employed over the past 50 years. Bone substitutes can be broadly categorized into bone grafts (autograft, allograft, xenograft) and the biomaterials can be titanium, ceramics (hydroxyapatite, TCP, calcium sulphate), natural polymers, synthetic polymers and many others ^4^. Work worldwide is focusing on the construction of bioactive, osteoconductive, biodegradable, bioabsorbable scaffolds in order to stimulate osteoprogenitor cells at the injury site without hyper stimulating unwanted cellular responses ^5^. 

There are many different types of polymers used in tissue engineering, each of which has completely different properties from other bone substitutes, and can be divided into biopolymers and synthetic polymers. Biopolymers of natural origin (collagen, gelatin, chitosan) are used for this purpose, as they mimic the structure and biochemical properties of the natural bone organic matrix, are able to stimulate a proper cellular response and function, on the other side it has some disadvantages, such as pure collagen scaffolds that remain their poor mechanical properties and pose a higher risk of infection and allergic reactions ^6^.

 A large number of tissue engineering materials based on synthetic polymers have been developed, including poly (ε-caprolactone) (PCL), polylactic acid (PLLA), polyglycolide (PGA), poly (lactide-coglycolide) (PLGA), poly (propylene fumarate) (PPF) and polyhydroxyalkanoates (PHA) ^4^ The benefit is that they improve healing without any foreign body residues remains. These polymers can be synthesized in large quantities under controlled conditions, ensuring uniform and reproducible properties while reducing the risks of infection and immunogenicity ^6^. Another important factor is how the body eliminates the remains of the biomaterial, in this case, biopolymers are degraded through simple hydrolysis, breaking the molecule into small units, and so their products can then be eliminated from the body through natural metabolic pathways, such as the citric acid cycle, or through renal excretion ^7, 8^.

Depending on the required application, PLLA has better physicochemical characteristics than other polymers. It also presents a diversification of applications, since simple changes in its physical and chemical structure can make it useful in different areas. Depending on the application, different products can be obtained using specific polymerization routes ^9^.

**Figure 1.**
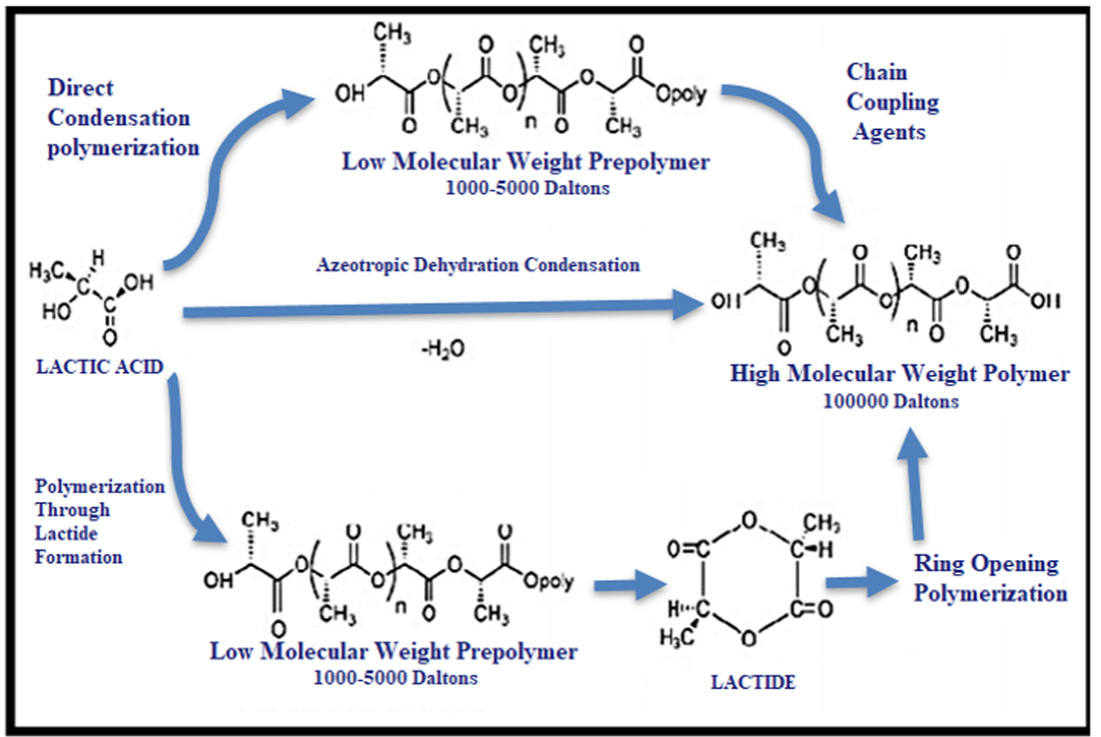
PLLA different synthesis methods.

Prior works done by our group ^10– 12^ have shown that the PLLA can be obtained using different routes ( Figure 1). In general, three methods can be used to produce high molecular mass PLLA of about 100,000 Daltons: (a) direct condensation polymerization; (b) azeotropic dehydrative condensation and (c) polymerization through lactide formation, the ring-opening polymerization. Currently, direct condensation and ring-opening polymerization are the most used production techniques.

While there are different ways to manufacture PLLA, its production is not easy. The synthesis of PLLA demands severe control of conditions such as temperature, pressure and pH, just as well as the use of catalysts and long polymerization time, which lead to a high-energy consumption to achieve the final product.

In addition to the intrinsic biomaterial characteristics previously mentioned, the cost of synthesizing or importing a specific polymer may affect its acquisition, application, or scientific study in some countries. The increase in the use of biomaterials around the world is evident and this expansion must be supported by strategies of biomaterial accessibility ^13^

**Graph 1.**
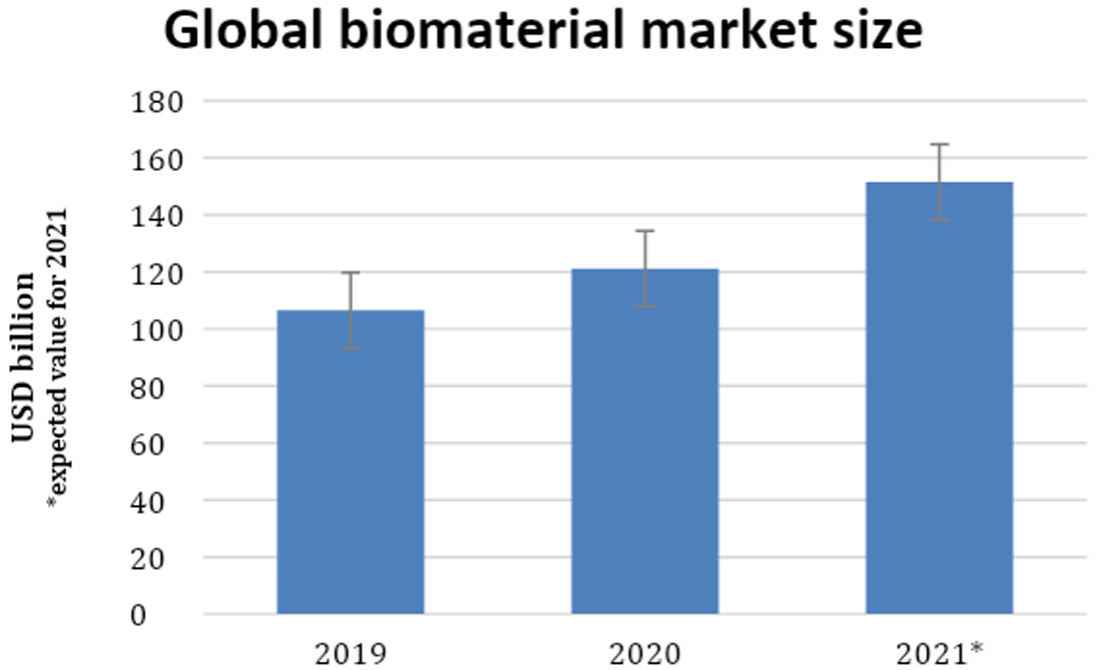
Global biomaterial market size 2019–2020 and expected value for 2021.

The global biomaterials market size was estimated at USD 106.5 billion in 2019, USD 121.1 billion in 2020, and is expected to reach USD 151.5 in 2021 as reported in the latest study by Grand View Research and polymers is the segment with the largest share in this statistic a participation of 40.8% in 2019.( Graph 1) ^14^

The data show that PLLA polymer has significant participation in the economic market of biomaterials, when analyzed the prices of biopolymer values are from USD 1800 to USD 3200 per kilogram of PLLA. In addition to the cost of the biopolymer as a raw material, packs of a few grams of PLLA for facial esthetic treatments cost USD 60 and a PLLA biofilm 300×300mm costs from USD 159 to USD 203 ^15, 16^.

One possible solution to reduce the costs of biomaterials studies is to import the monomer and then perform the polymerization, this strategy allows the material to be synthesized in a cheaper and more accessible way, thus opening the possibility to learn more about the material and discover new ways of polymerization and even to obtain its monomer.

Based on the above mentioned strategy, after studies, the national PLLA polymerization was produced and the results published in previous works ^10^, subsequently hemocompatibility and cytotoxicity studies proved the in vitro biocompatibility of the material, in order to verify if it could have a medical application. These studies showed that the strategy of elaborating a polymerization from imported monomers was viable and that the PLLA biopolymers produced could be an accessible solution for use ^11^.

The *in vivo* biocompatibility study is a crucial requirement to prove that the polymer manufactured will have the same medical applicability as the biomaterial polymerized elsewhere. When referred to biocompatibility analysis means the ability of a material to produce an appropriate host response when applied as designed, and it is this response that must be evaluated ^17^.

This work evaluates the *in vivo* biocompatibility of biomaterial synthetized from Lactide monomer polymerization, showing the possibility of synthesis at low cost with a quality final product that can bring real benefits to research and the application of its results in the health of parents with less financial possibilities for scientific research.

### Study objective

This work aims to evaluate the *in vivo* biocompatibility of biomaterials synthesized from the polymerization of Lactide monomers and compare the final synthesis cost to the PLLA biopolymer import price.

## Methods

This study has been reported using CONSORT guidelines and ARRIVE guidelines.

### Manufacturing of PLLA

***Poly-lactic acid synthesis.*** The synthesis of PLLA was conducted by bulk polymerization by adding L-lactide monomer into a glass reactor containing the catalyst Sn (Oct) 2 (Sigma). The proportion monomer/catalyst was 0.5%. The mixture was immersed in an oil bath at 140°C for 2 hours under nitrogen flow. The produced polymer was dissolved in chloroform, CHCl3 (Merck), precipitated in ethyl alcohol and dried in a vacuum oven at 60°C for 12 hours. After the synthesis were manufacture tablets of PLLA.

**Figure 2.**
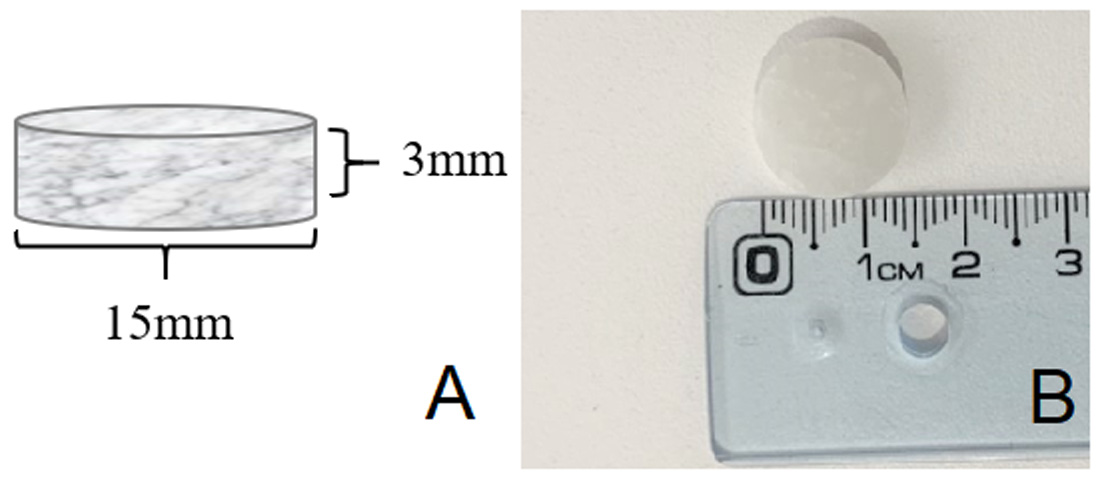
**A** – 3D representative image and dimensions of the PLLA tablet. **B** - photo of a compacted PLLA pellet, ready for insertion into a rabbit lesion.

***Manufacturing PLLA tablet.*** The scaffold size was determined by reviewing previously published studies that determine the size of critical lesions for osseointegration and biocompatibility studies in rabbits ^18, 19^. PLLA tablets ( Figure 2) of 3mm × 15mm were produced by compression of the polymerized raw material under a hydraulic press.

### In vivo experiments

***Study design.*** The animals were selected and divided into groups, at the post-surgical time determined for each group the initial number of animals was not changed as shown in the study flow diagram.

***Experimental models - eligibility criteria.*** 12 New Zealand female rabbits (age = 16–20 weeks) were assigned to two groups:(1) Injury + PLLA group (n = 6), (2) Injury no PLLA group (n = 6). Each group was divided into two subgroups at six and nine months post-surgical procedure. Rabbits were kept in individual cages with food (PROVIFE, Argentina) and water ad libitum. All procedures were made according to the approval of the ethics committee nº44371/0028 of 1MED423- University National of Rosary, Argentina. Their regulations are in accordance with well-established guidelines for the care and handling of animals to minimize animal pain and suffering according to the 3Rs (replacement, reduction and refinement) and follow international regulations for the care and use of laboratory animals. All efforts were made to ameliorate harm to animals

***Pre-surgical preparation.*** Antibiotic prophylaxis and anesthetic treatment were performed. Before the surgical procedure, rabbits received antibiotic prophylaxis Cefazolin 50mg/Kg/day (administered intramuscularly). Anesthetic treatment was performed using a combination of two drugs administered intramuscularly: ketamine hydrochloride 35mg/kg animal, xylazine hydrochloride 2% 18mg/kg animal, and acepromazine maleate 1mg/kg animal. The anesthetic effect lasted 45 – 60 minutes.

***Surgical procedure.*** The surgeries were performed under aseptic conditions. A longitudinal incision of 3 cm total length was made parallel to the sagittal suture below the Lambdoid suture (5–6 cm long). The central perfuration point is marked with a surgical marker, the drilling was done with a 15 mm drill, made of stainless steel with laser engraving, generating a hole of 15 mm in diameter and +/-3 mm in depth. After dissection of the periosteum, the defect was made in the center of the sagittal suture, 2 mm below the Lambda suture. In one group of animals PLLA was introduced in the center of the lesion (Lesion + PLLA Group) and in the other group the bone lesion was maintained (Lesion without PLLA Group) ( Figure 4).

***Post-surgical procedure.*** The rabbits were clinically monitored daily in basis as to overall status, mobility and food intake. Post-surgical analgesia was performed using tramadol every 12 hours, (6 mg / kg / day) intramuscularly during three days. Antibiotic cephalexin, 50mg/kg/day and anti-inflammatory meloxicam 0,75mg/kg/day intramuscularly on the day of surgery and 3 days following surgery.

### Biochemistry studies

***Hemogram.*** Blood samples were collected before sacrifice. The blood samples were collected in EDTA tubes to perform hemogram, after homogenization of the samples. Red blood cells (RBC), white blood cells (WBC), hemoglobin (Hb), hematocrit (Hto), mean corpuscular volume (MCV) and platelets were evaluated.

***Transaminases.*** The serological study was performed on blood samples without anticoagulant, centrifuged for 10 minutes at 10,000 rpm, to separate the serum fraction from the blood, where the values of the enzymes transaminases, glutamate-oxalacetic transaminase (GOT) and glutamate pyruvate transaminase (GPT) were determined. All determinations were performed with Wiener lab Kids (Argentina).

Biochemical statistical evaluation was performed with non-parametric analysis of variance (Wilcoxon). A p value <0.05 was considered significant

### Animal euthanasia and sample collection

The animals were euthanized in the 50 % of each group at six-month post-surgery and the others at nine months, with an intraperitoneal injection of five times the value of the anesthesia dose. Then, cranial were obtained by axially cutting the neck near the base of the skull with a bench saw, which were immediately used for tomographic, macroscopy and histology studies.

### Macroscopy observation

Prior to sacrifice, each implanted area was inspected. Surface characteristics such as skin color, scarring, edema, erythema, and elevations were evaluated. A sagittal incision was then made at the scar site and the lesion area was cut with a 5 mm margin from the edges to remove a portion of bone with the central lesion. After removal of the skull fragment containing the central lesion, the endocranial and exocranial side of the fragment was evaluated for edema, surface characteristic, implant position, exudate or other signs of infection.

### Tomographic studies

Tomographic studies were performed on the day of sacrifice of the group of animals, each skull obtained from the axial section was immediately used for tomographic study with a 16-channel Toshiba multislice CT scanner for qualitative analysis of the characteristics of the lesion edge and the progression of the tissue around the biomaterial.

### Histological analysis

After euthanasia, tissue preparation was conducted according to a previously described method. Briefly, the lesion area was explanted with a 5 mm margin around, fixed in 4% formaldehyde for 24 hours and then dehydrated in a series of increasing concentrations of alcohol and xylene. After that, the samples were serially cut (5 µm thick) using a manual rotary microtome (Micron-Zeiss, Germany), and stained with hematoxylin and eosin and by Masson trichrome staining. All samples were examined by light microscopy and evaluated. Photomicrographs were taken from slides of each specimen using a digital camera mounted on an Olympus CH30 microscope.

### Osteoregenerative analysis

Studies were conducted to consider if the proportion of regenerated bone in animals in the group implanted with the scaffold under study was greater relative to animals in the group not implanted with PLLA at each of the time points considered for this experiment, six months and nine months, respectively.

Three histological sections were studied for each animal in each of which three sites were randomly selected. ImageJ software was used for the observation of de novo regenerated bone. The New bone formation was quantified and expressed as a percentage of total bone (% BV/TV) in the area surrounding the lesion.

Data were analyzed using linear mixed models implemented in Infostat software. Outliers were removed before the analysis was performed.

Statistical analyses: The variable of interest is worked out in original scale, in proportion between 0 and 1. To detect significant differences between Groups, linear mixed models ^20^ were fitted and the Bayesian information criterion ^21^ was used to select the best fit. Fisher's a posteriori test was used with a significance level of 0.05. The software used for the analysis was Infostat ^22^. These procedures were followed for both the evaluation at six months and nine months and between six months.

### Production and import cost analysis

**Figure 3.**
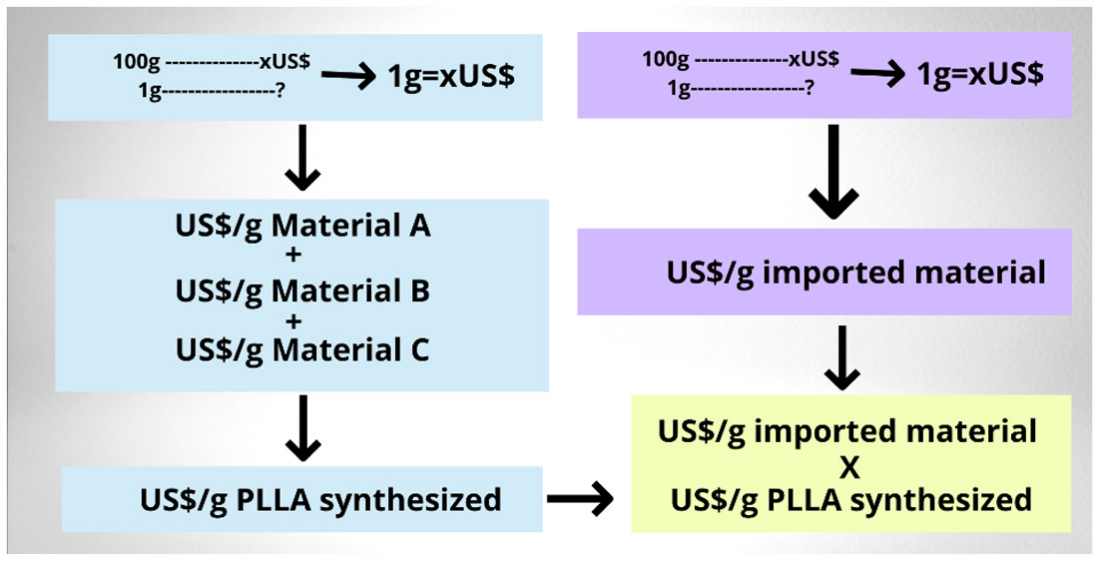
Blue square representing cost calculations of PLLA synthesis. Violet square representing cost calculations of each gram of already synthesized imported PLLA. Yellow square represents comparison of costs of laboratory synthesized material and imported biomaterial.

The cost of the raw material needed to synthesize the biomaterial from the lactide monomer was compared with the cost of importing the PLLA polymer. The currency conversion of the prices in dollars (US$) to real (R$) was performed. To analyze the cost of synthesizing 1 gram of PLLA, the price of 100g of lactide monomer, dudecanol, octanoate and the Nitrogen (N2) cylinder was obtained, and then the cost of each quantity used in the synthesis was determined. The costs of each material were added up to obtain the price of 1g of synthetized polymer. This value was compared to the cost of 1g of the imported material by dividing the price of the material by the content of 100 grams of the package. ( Figure 3)

## Results

### Manufacturing of PLLA- Poly-lactic acid synthesis

The PLLA was synthesized by opening of the cyclic dimer of L-lactide in order to obtain high molecular weight polymer. The synthesis temperature was maintained at 140 °C/ thus avoiding, high temperatures, which lead to a depolymerization process which allows the decrease of the molecular weight of the polymer ^23^.

The obtained polymer had the PLLA molecular weight 86.93 g/mol as mentioned in previous works ^10^.

### Surgical Procedure

**Figure 4.**
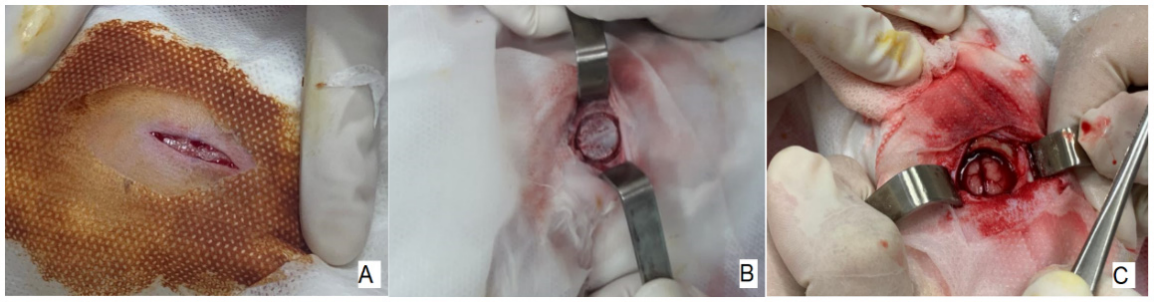
Surgical Procedure. A 3-cm full-thickness longitudinal incision ( **A**). The central drilling point ( **B**). Defect in the center of the sagittal suture, 2mm below the Lambda suture ( **C**).

As can be seen in Figure 4, the surgical procedure with the selected drill allowed the achievement of a central lesion with a diameter considered as a critical lesion, and it was also possible for the biopolymer to be placed in the center in order to analyze its biocompatibility with the edge of the bone in question in the group (Injuria +PLLA).

In Figure 4-A, after asepsis with iodopovidone and covering the surgical area on top of the head, a sagittal cut was made with a scalpel. After removal of the periosteum, the start of the circumferential lesion can be seen through a 15mm trephine, before the central bone is removed ( Figure 4-B). After total removal of the central bone, a part of the brain can be seen in Figure 4-C, covered by the meninges.

### Biochemestry results

**Figure 5.**
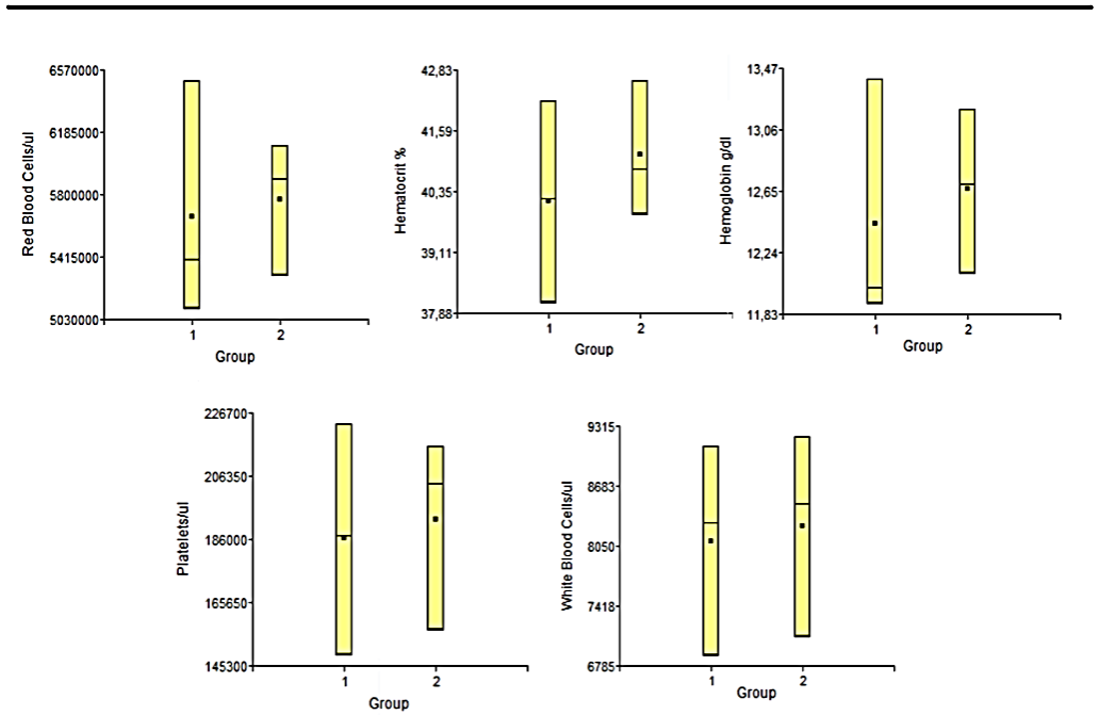
In this figure we have the graphs representing the values of the different variables measured in the blood count study of the animals under study, in the period of 6 months post-surgery. 1: Lesion + PLLA group. 2: Lesion No PLLA group.

**Figure 6.**
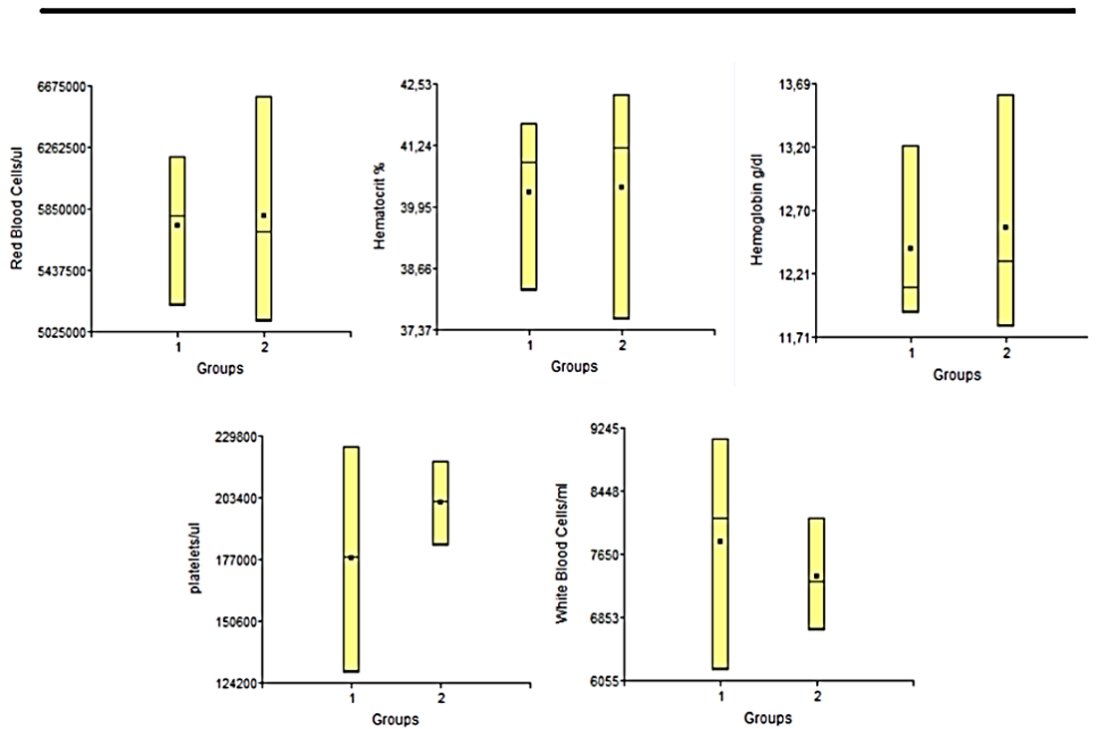
In this figure we have the graphs representing the values of the different variables measured in the blood count study of the animals under study, in the period of 9 months post-surgery. 1: Lesion + PLLA group. 2: Lesion No PLLA group.

***Hemogram.*** The hemogram statistical studies showed that there were no significant differences between the groups at any of the times studied, in any of the variables considered. In Figure 5 and Figure 6 we can observe the results obtained the day before euthanasia. Each of the results obtained for each variable applied to each sample, were always within normal ranges for animals of this line and age ^24^

**Figure 7.**
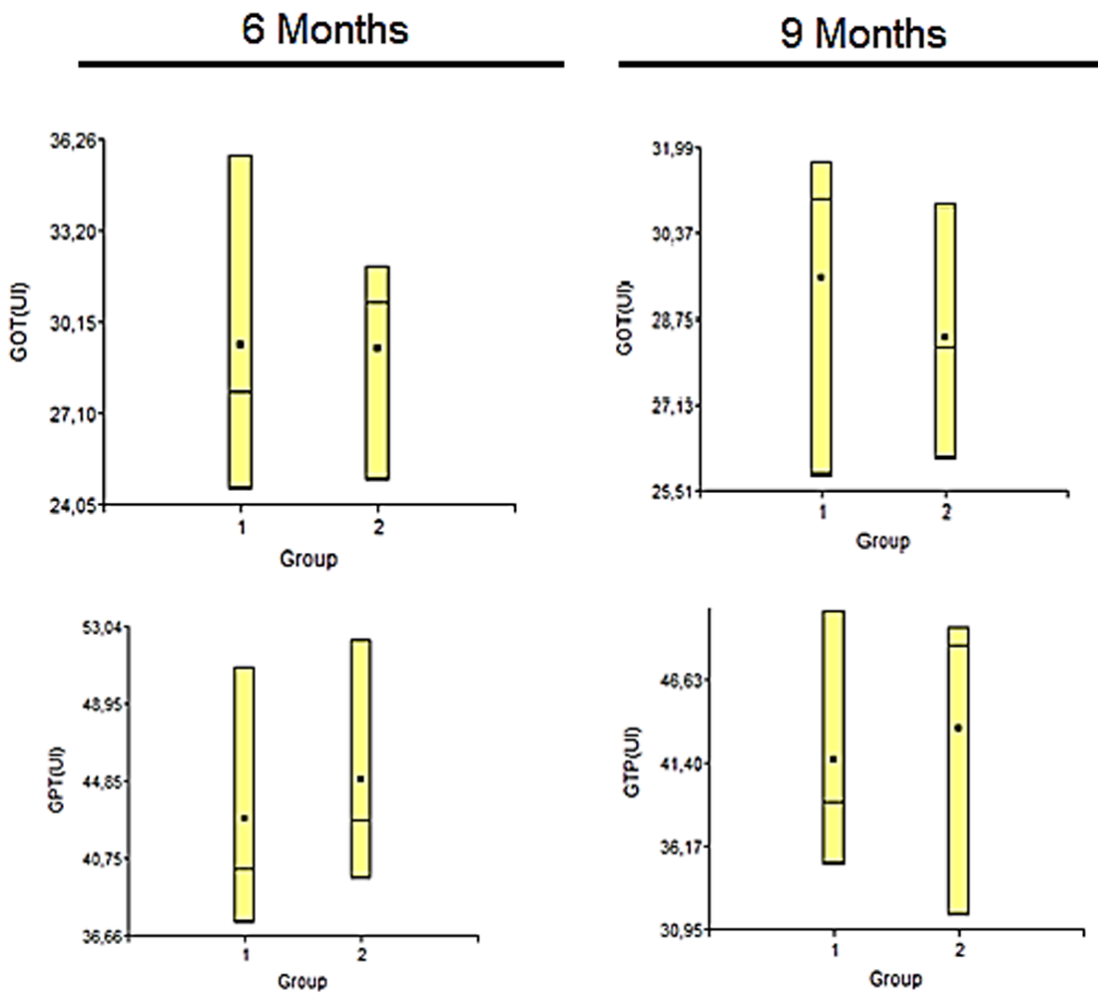
In these graphs are represented the values for the different variables measured in the transaminasas study of the animals under study, in both period post-surgery 6 and 9 months. Group 1: Injury No PLLA. Group 2: Injury + PLLA.

***Transaminases.*** Statistical studies of transaminase levels showed that there were no significant differences between the groups for any of the variables considered. Each of the results obtained for each variable applied to each sample ( Figure 7).

### Tomographic results

The images observed on the CT scans allowed an analysis of the lesion border in the Injury+PLLA group and the Injury No PLLA group.

**Figure 8.**
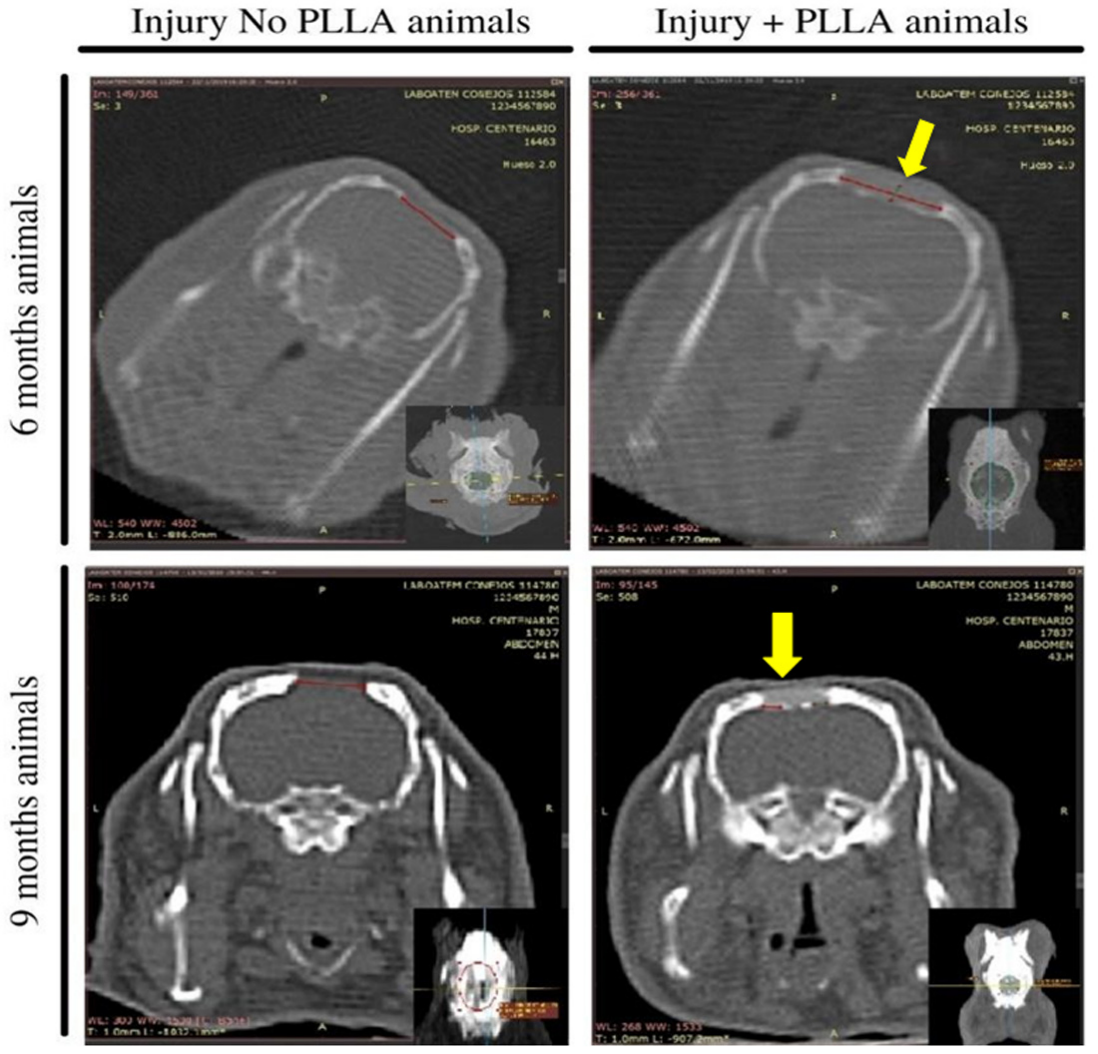
Representative tomographic images of animals at 6 and 9 months post implantation. The larger images represent a coronal slice and the smaller images in the lower right corner represent axial cuts. On the left images, represent the animals that did not receive the PLLA implant, where the red line shows the lesion area. The right images represent animals that received PLLA implantation in the center of the lesion (yellow arrow) and the red line shows the lesion area.

In the Injury No PLLA group at 6 and 9 months, it is possible to observe the formation of a small fragmented layer of bone tissue over the meninges, however, there are no differences in bone formation activity at the edge of the lesion. The analyses of the animals that have the PLLA implant in their lesion, showed hyperdense borders with signs of bone regeneration. In addition, it can be seen that under and above the implant there are hyperdense sectors, also indicating the formation of a layer of bone tissue around the implanted matrix. ( Figure 8)

### Macroscopy observation results

**Figure 9.**
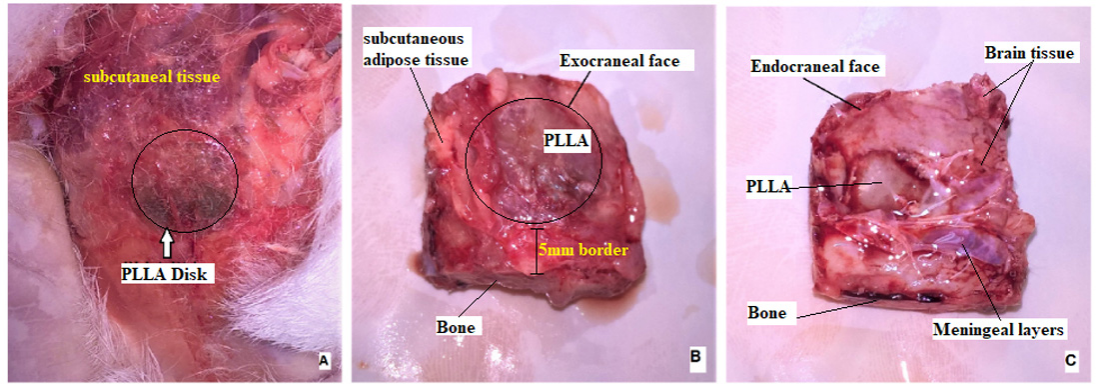
Cranial gross appearance of the defects, showing the fibrous scar covering the defect area ( **A**), exocranial ( **B**) and endocranial face ( **C**).

After sectioning the area with the central lesion, a clinical presentation of the groups examined at six and nine months is shown in Figure 9. A fibrous scar covered the entire area of the defect. From the Exocraneal view much of the polymer can be visualized below a thin layer of subcutaneous tissue. In the endocranial view it was covered by the adhered meninges, where the biopolymer could hardly be assessed. No apparent indications of infection were detected in any of the experimental groups macroscopically.

### Histological results

***Hematoxilin and eosin.*** In the hematoxylin and eosin staining results, at a 10X magnification it is possible to analyze the cell types present at the margin of the lesion and compare them with the group of animals that did not receive the PLLA biopolymer implant. In the six-month-old animals without implantation, it is possible to observe the scarce formation of connective tissue and some inflammatory cells and the irregular and undefined border of the lesion.

**Figure 10.**
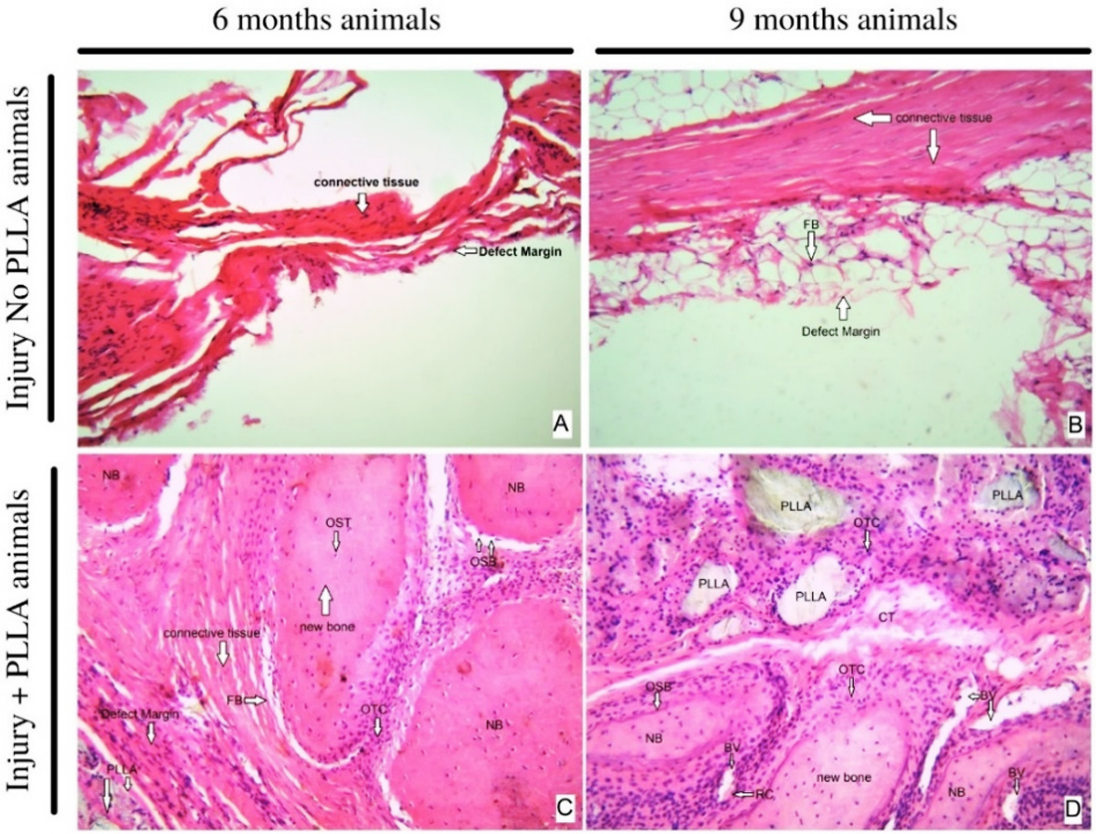
HE staining- original magnification 10x – **A** 6 months, Injury No PLLA animal. **B** nine months, Injury No PLLA animal. **C** six months, Injury + PLLA animal. **D** 9 months, Injury + PLLA animal. Bone is presented as a compact structure in a pink color, connective tissue in light pink and the scaffold structure (PLLA) in light grey. OSB, osteoblast; OTC, osteoclast; OST, osteocyte; FB, fibroblast; BV, blood vessel; NB, new bone and Defect Margin indicates edge the tissue boundary.

When analyzing the six-month-old animals with bone implant, there is a thick layer of connective tissue at the margin of the biomaterial, with an evident and well-defined border. In the region of contact with the polymer, groups of inflammatory cells can be seen around the fragments of biomaterial. In the surrounding connective tissue, besides fibroblasts, it is possible to see bone tissue formation, with the presence of osteoblasts, osteocytes and osteoclasts. ( Figure 10 – A and C)

When comparing and analyzing the nine-month-old animals, it is possible to verify that the animals with a critical lesion without implant have an increased layer of connective tissue around the edge of the lesion, the presence of adipose tissue, and few inflammatory cells. In the animals nine months old with implants, the plaque fragments are surrounded by phagocytic cells, surrounded by abundant connective tissue and new bone tissue, and abundant osteoblastic and osteoclastic cells can be seen. Blood vessels are also present and distributed around the PLLA biopolymer. In none of the animals is there the formation of thick connective tissue suggestive of fibrous capsule. ( Figure 10 – B and D)

When analyzed at 40x magnification, the cell types present at the edge of the lesion are observed. The six-month-old animals with lesions without polymer have the formation of abundant dense connective tissue, without signs of intense inflammation or signs that indicate regenerating cellular activity such as the presence of new bone tissue. When compared to the six-month-old animals, at the same 40x magnification, we see the presence of inflammatory tissue surrounded by fragments of polymer and also abundant blood vessels throughout the area.

### Masson's trichrome

The collagen is seen in blue or greenish color, while the bone acquires a red color. through this differentiation we can delimit the color margin in the ImageJ program, quantifying the percentage of bone present at the edge of the lesion and comparing the different groups and times.

After Masson staining and quantification of the area in pixels represented by dark blue and red colors, the area of bone growth bordering the lesion can be seen. In a qualitative analysis of the Masson staining, it can be inferred, together with what was observed in the hematoxylin and eosin staining, that the animals implanted with PLLA present a greater amount of bone tissue surrounding the lesion.

**Figure 12.**
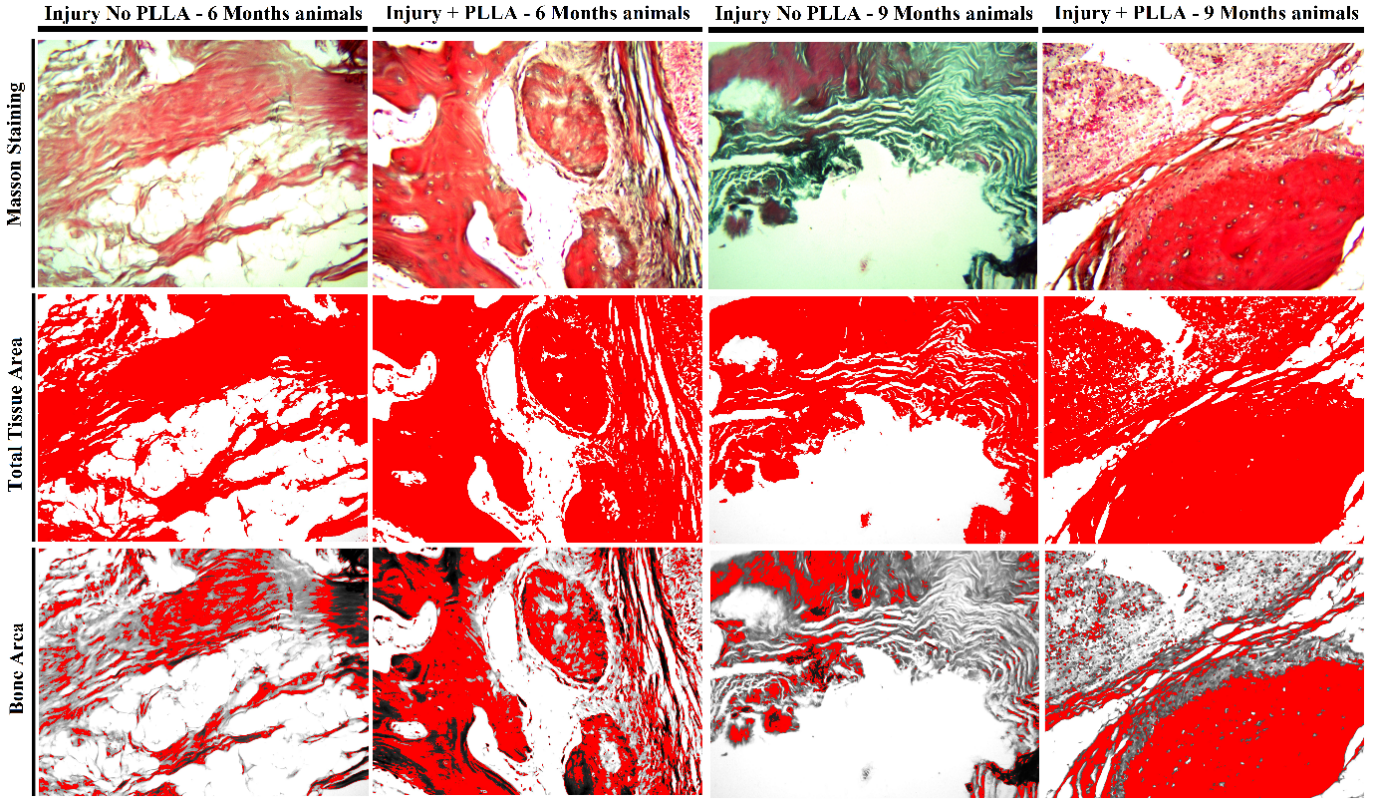
Masson Staining - original magnification 10x. -Histomorphometric analysis of connective tissue lesion area and bone tissue area in six- and nine-month-old animals with and without PLLA. First row with original photos at 10X magnification with Masson staining. Second row with total tissue area in red by image analysis software image J. Third row representing bone tissue area in red.

Together with the images, we can analyze below the statistical data that represents the area of bone and connective tissue of the analyzed groups ( Figure 12)

### 1. Osteoregenerative statistical results

**Graph 2.**
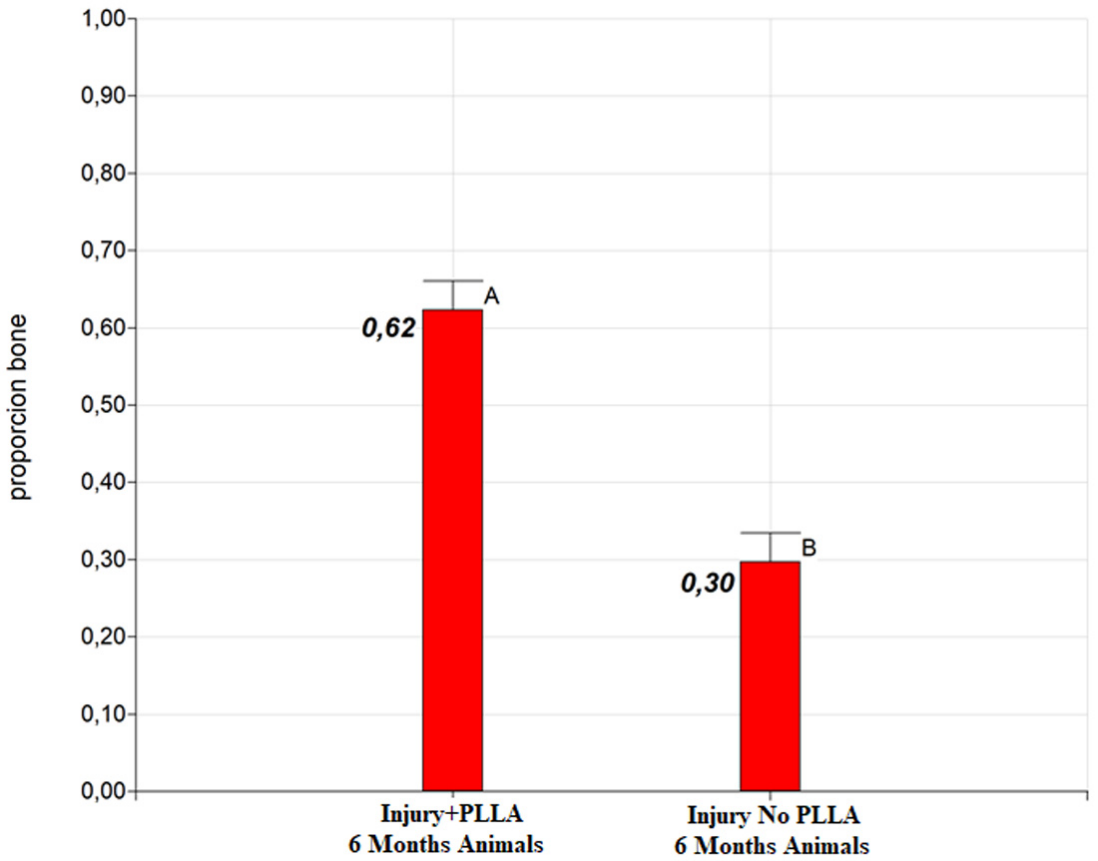
Difference of adjusted means for treatments: PLLA 6 month and Control 6 month. Different letters indicate significant differences in LSD Fisher test with a significance of 0.05.

**Graph 3.**
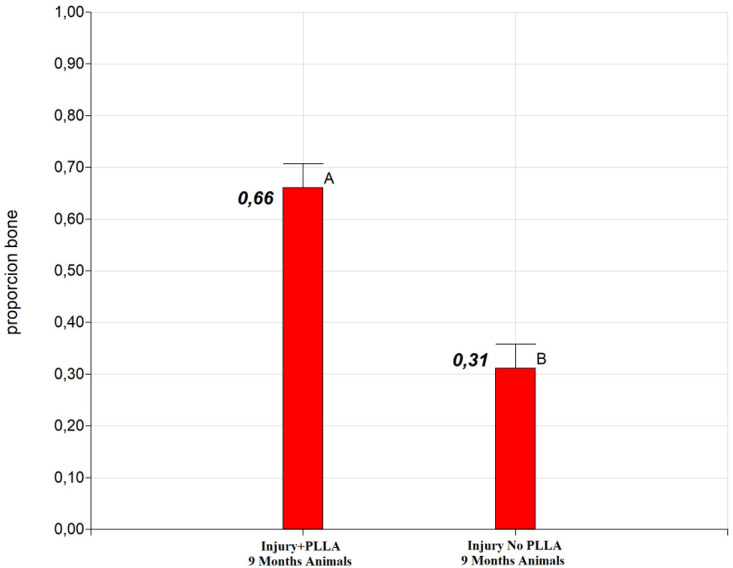
Difference of adjusted means for treatments: PLLA 9 month and Control 9 month. Different letters indicate significant differences in LSD Fisher test with a significance of 0.05.

Significant differences were detected between the groups at both six and nine months of post-surgical evaluation. The Injury+PLLA group achieved 62% bone regeneration at six months, while the Lesion+PLLA group achieved only 30% bone regeneration ( Graph 2). At nine months of evaluation, the Injury+PLLA Group achieved 66% of both regenerations, compared to 31% in the Injury No. PLLA Group ( Graph 3).

### Production and import cost analysis results

The prices of the raw material for the polymerization of the biomaterial in the laboratory were obtained through quotations in Sigma Aldrich. and Corbion.


$$a)\;Lactade\;monomer\;cost=\frac{377950}{1000}=\text{R}\$377,95$$

$$b)\;Sn\;(Oct)\;2\;cost=\frac{1059}{1000}=\text{R}\$1,06$$

$$c)\;CHCl3\;(1Lcost=\frac{0,07866}{1000}=\text{R}\$0,00008$$

$$d)\;N2\;cost=\frac{429}{10}=\text{R}\$42,90$$

e)Polimerization50gcost=377,95+1,06+0,00008+42,90=421,91

$$f)\;1g\;cost=\frac{421,91}{50}=\text{R}\$8,43$$

The price of synthesizing 50g of PLLA was first calculated by dividing the value of R$7. 559.00 per 50 grams of the lactide monomer (1kg) Purasorb L, R$1413.00 of the Tin Octanoate (1kg) divided by the 0.75 grams needed, R$138.00 of the CHCl3 (1L) cost was divided by the 0.00057ml used and the nitrogen used for 150 minutes of reaction corresponds to 3 liters, where the cost per liter of N2 was calculated by dividing the R$143.60 by the 10L and then multiplied by the 3 liters needed. Once the cost of producing 50 grams of polymer was obtained, this cost was divided by the value of 50 and the cost for each gram of polymer synthesized in the laboratory was found ( Table 1).

**Table 1.** The prices of the products used for the synthesis of the biomaterial are expressed above.

Material	R$ Price*
**L-lactide monomer Purasorb L (1kg)**	R$ 7.559,00
**Sn (Oct) 2 (1kg)**	R$1.413,00
**CHCl3 (1L)**	R$138,00
**N2 (10L)**	R$143,6


$$a)\;TCI\;America^{TM}1g\;cost=\frac{287,28}{25}=\text{R}\$11,49$$

$$b)\;ACROS\;Organic^{TM}1g\;cost=\frac{348,03}{25}=\text{R}\$13,92$$

The price of the imported PLLA polymer was quoted by two companies: TCI America and ACROS Organics. The packages contained 25g of PLLA polymer each. The cost per gram of imported polymer was calculated by dividing the package price by 25, where the costs found for each brand were: R$11.49/g of PLLA polymer (L-Lactide 98.0+%, TCI America™) and R$13.92 of the polymer (L-Lactide, 98%, ACROS Organics™) ( Table 2).

**Table 2.** Prices of PLLA polymers imported from different brands - TCI America and ACROS Organics. *The dollar values were converted to real with WestenUnion quotation of August 05, 2021 where 1US$= R$5.223.

Material	Price	R$ Price *
**L-(-)-Lactide 98.0+%, TCI America™** **(25g)**	US$ 55,00	R$287,28
**L-Lactide, 98%, ACROS Organics™** **(25g)**	US$ 66,63	R$348,03

Comparing the costs for each imported gram of PLLA of R$11.49 and R$13.92 to the obtained value of R$8.43 for laboratory synthesized PLLA, a difference of R$3.06 is calculated between synthesized PLLA and imported PLLA (TCI America™) and a difference of R$5.49 for laboratory synthesized PLLA compared to imported PLLA (ACROS Organic™).

## Discussion

From the work performed, it was possible to synthesize the polymerization of PLLA from lactide monomers using the cyclic ring opening technique and subsequently create implants with the exact size and shape of the lesion to be repaired. The implants developed had characteristics that allowed them to be easily implanted perfectly into the lesion site by means of simple manipulation. This makes the material obtained a potential scaffold that can be synthesized in a three-dimensional way and be applicable for different injury shapes. 

As published by other authors, implantation of a biomaterial could induce a host reaction to the implant that determines the integration outcome and the biological performance of the implant ^25^. Based on these statements, clinical observations of the animals were performed and the results provided evidence that there were no inflammatory events. In this work it is very important to remark the hemogram monitoring level of the implanted animals, compared to the injured animals that did not receive the implant, since this allows to evaluate some syntemic responses. The normal red blood cell values did not suggest hemolysis, and the white blood cell parameters remained within normal ranges.

Another important study when dealing with bioabsorbable biomaterials is to analyze transaminases, indicators of possible hepatoxicity, because the degradation of polymers into their respective monomers can give rise to the systemic circulation of these biomolecules, requiring hepatic biotransformation for waste disposal ^26^. The terminology used for the word biodegradable is often misapplied as a synonym for bioabsorbable in many literatures. It is important to infer that biodegradation does not mean that the material will leave the organism, it will just lose its primary conformation. When we mention the word bioabsorption, we are referring to the power of metabolization or excretion that the body has in relation to this biomaterial.

Thus, the analysis of transaminases allows us to evaluate the co-participation of the liver and verify whether it may be suffering damage from the presence of systemic circulation of biomolecules ^27^. Therefore, it was important that in the experimental model presented in this work, the variables of both transaminases were not altered, without significant intergroup differences, with values within the range of animals of the same line and age, reinforcing the primary idea of biocompatibility without systemic damage by biodegradation.

When it comes to analyzing the degradation surface of the biomaterial it is important to take into account that PLLA has a degradation time ranging from 6–24 months, depending on temperature, ph, humidity and others ^28^. This means that the period covered by the current study is limited in data to make any statement in this respect.

Thus, the analysis of the lesion site through tomographic images were qualitative ( Figure 8) showed that the biomaterial allows bone regeneration in a centripetal way, performing the osteointegration process from the edge of the lesion towards the biopolymer. In animals without PLLA implantation, no significant change in the lesion was verified.

The inflammatory process occurs in phases, and it is important to analyze the macroscopic site of injury, looking for signs of inflammation, such as fibrotic capsule formation, and any indicator of biocompatibility failure. In the macroscopic evaluation ( Figure 9), no signs indicative of regenerative failure or exacerbated inflammatory response were observed. This information is in accordance with other published papers that affirm that several factors are taken into consideration in a biocompatibility study. We know that the insertion of a biomaterial can naturally produce a local inflammatory response with attempts to eliminate the inflammatory agent and regenerate the affected tissue. However, this inflammatory response must be within expected limits at each post-injection time point ^29^.

In biocompatibility studies, performing histological analysis is crucial to understand the interaction of the implant site with the implanted polymer. The interaction between the host immune system and the implanted material depends on the tissue surrounding the implant, which will drive tissue-specific innate defenses and the following induction of adaptive immune responses, so analyzing the type of cellularity is indicated ^30, 31^.

In this work, histological studies allow us to analyze the type of cell present at the edge of the lesion. We can infer that, in the absence of biomaterial, the critical lesion evolves to a sclerotic border. In the animals ( Figure 10) that received the biomaterial, not only showed biocompatibility and absence of fibrotic capsule, but also showed formation of connective repair tissue and the beginning of bone tissue formation, which can be confirmed with further staining and analysis

**Figure 11.**
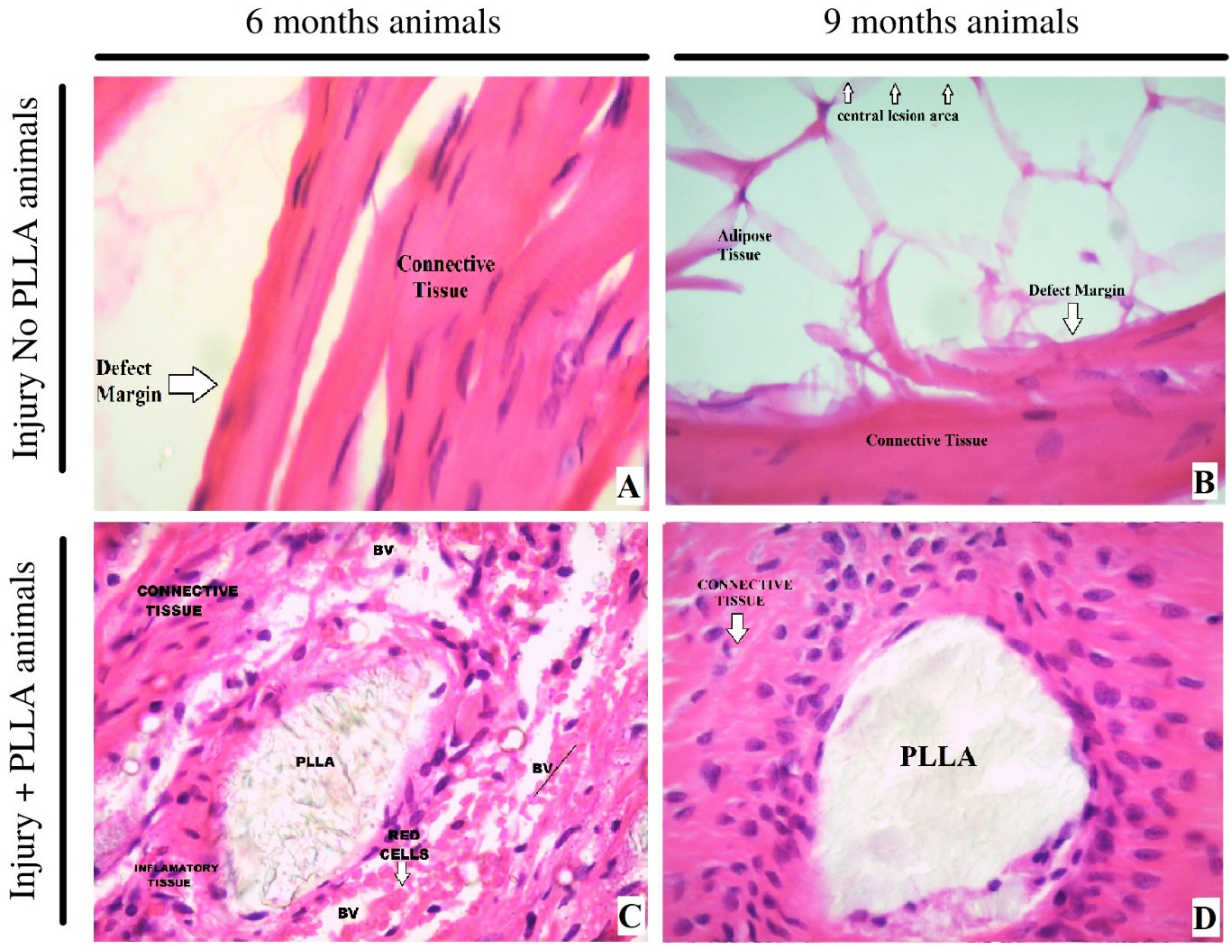
HE staining- original magnification 40x. – **A** 6 months, Injury No PLLA animal. **B** 9 months, Injury No PLLA animal. **C** 6 months, Injury + PLLA animal. **D** 9 months, Injury + PLLA animal. Bone is presented as a compact structure in a pink color, connective tissue in light pink and the scaffold structure (PLLA) in light grey. BV – Blood Vessel. Defect Margin indicates edge the tissue boundary.

Knowing that the inflammatory response changes its profile over time and according to the type of biomaterial present, taking weeks or even months for an acute response to change to a chronic one ^32, 33^, we seek to verify a difference between the tissue profile in animals six and 9 months post-surgery. it is evident that when analyzing the margin and the center of the lesion at 40x magnification in the animals nine months without implantation, the center of the lesion was taken by adipose tissue, the border contains connective tissue and little sign of neovascularization or bone regeneration, the cells present indicate a process of repair of the border without possible regeneration of the lesion. When the center and the edge of the lesion are checked in the animals with implanted polymer, the initial acute inflammatory process has given way to the degradation of the polymeric matrix and the connective tissue surrounding the polymer is occupied by bone tissue cells. In the center of the lesion, islands of biomaterial fragments can be seen, completely surrounded by a new bone matrix, as shown in Figure 10 and Figure 11.

The PLLA implant promoted a very significant increase in the percentage of bone tissue formation. This would reinforce the proposal to promote our material in the field of regenerative medicine, since it would not only show biocompatibility characteristics, but would also promote bone tissue regeneration in a gradual manner.

Analyzing the strong economic impact nowadays generated by biomaterials and their very important applications in the medical sector, one can verify that any strategy that seeks to reduce their cost and simplify their synthesis process will be considered a mechanism to promote the accessibility to biomaterials.

Through the cost analysis mentioned, the synthesized biomaterial has a difference of $3.06 between synthesized PLLA and imported PLLA (TCI America™) and a difference of $5.49 for laboratory synthesized PLLA compared to imported PLLA (ACROS Organic™) Per gram of biomaterial. Confirming that the synthesized biomaterial, besides completing with the expected biocompatibility requirements, also proved to be cheaper than the import process.

## Conclusion

This study proved that ring-opening polymerization is a feasible process for the production of lactic acid-derived PLLA, the results demonstrated that the synthesized polymer presents the characteristics of biocompatibility and osteoregenerative potential, as well as being a low-cost and accessible procedure, as an initial proposal to become cheaper.

## Data availability

### Underlying data

Zenodo: ARRIVE Questionnaire, CONSORT Check-list and Flow Diagram + TGO, TGP, HEMOGRAMA AND OSTEOREGENERATIVE DATA - Paper: "Biological performance of a bioabsorbable Poly (L-Lactic Acid) produced in polymerization unit: in vivo studies", https://doi.org/10.5281/zenodo.5396921 ^23^. 

This project contains the following underlying data:

- Biochemistry transaminases Data - GOT & GTP.xlsx
- Data - Hemogram 6 months animals.xlsx
- Data - Hemogram 9 months animals.xlsx
- Osteoregenerative Masson Staining bone area.xlsx

Zenodo: Images, graphs and tables from the article: Biological performance of a bioabsorbable Poly (L-Lactic Acid) produced in polymerization unit: in vivo studies - https://doi.org/10.5281/zenodo.5216707.

This project contains the following underlying data:

- TAC CONEJO 41 - 6 MESES+PLLA600.tif
- TAC CONEJO 44 - 9MESES CONTROL600.tif
- TAC CONEJO 45 - 6 MESES CONTROL600.tif
- TAC CONEJO 45 - 9MESES +PLLA600.tif

Zenodo: Biological performance of a bioabsorbable Poly (L-Lactic Acid) produced in polymerization unit: in vivo studies - HEMATOXYLIN&EOSIN, MASSON AND CT SCAN IMAGES, https://doi.org/10.5281/zenodo.5396584.

### Reporting guidelines

Zenodo: ARRIVE Questionnaire, CONSORT Check-list and Flow Diagram + TGO, TGP, HEMOGRAMA AND OSTEOREGENERATIVE DATA - Paper: "Biological performance of a bioabsorbable Poly (L-Lactic Acid) produced in polymerization unit: in vivo studies", https://doi.org/10.5281/zenodo.5396921. 

This project contains the following reporting guidelines:

- File 1 CONSORT 2010 Checklist - PLLA.pdf
- File 2 ARRIVE Compliance Questionnaire - PLLA Animals.pdf

Data are available under the terms of the Creative Commons Attribution 4.0 International license (CC-BY 4.0).

## References

[1] Stratakis E : Novel Biomaterials for Tissue Engineering 2018. *Int J Mol Sci.* 2018;19(12):3960. 10.3390/ijms19123960 30544860 PMC6321414

[2] Bhatt RA Rozental TD : Bone graft substitutes. *Hand Clin.* 2012;28(4):457–68. 10.1016/j.hcl.2012.08.001 23101596

[3] Campana V Milano G Pagano E : Bone substitutes in orthopaedic surgery: from basic science to clinical practice. *J Mater Sci Mater Med.* 2014;25(10):2445–61. 10.1007/s10856-014-5240-2 24865980 PMC4169585

[4] Arner JW Santrock RD : A historical review of common bone graft materials in foot and ankle surgery. *Foot Ankle Spec.* 2014;7(2):143–51. 10.1177/1938640013516358 24425807

[5] Cicero AM Issa JPM Feldman S : Matrices de tercera generación en la ingeniería de tejidos óseos. Asociación Argentina de Osteología y Metabolismo Mineral; Actual Osteol.2017;13(2):157–176. Reference Source

[6] Thrivikraman G Athirasala A Twohig C : Biomaterials for Craniofacial Bone Regeneration. *Dent Clin North Am.* 2017;61(4):835–856. 10.1016/j.cden.2017.06.003 28886771 PMC5663293

[7] Santos AR Jr Wada MLF : Bioreabsorbable polymers for cell culture substrate and tissue engineering. *Polímeros: Ciênc Técnol.* 2007;17(4):308–17. 10.1590/S0104-14282007000400010

[8] Middleton JC Tipton AJ : Synthetic biodegradable polymers as orthopedic devices. *Biomaterials.* 2000;21(23):2335–46. 10.1016/s0142-9612(00)00101-0 11055281

[9] Fukushima K Kimura Y : An efficient solid-state polycondensation method for synthesizing stereocomplexed poly(lactic acid)s with high molecular weight. *J Polym Sci Part A-Polym Chem.* 2008;46(11):3714–3722. 10.1002/pola.22712

[10] Lopes MS Jardini AL Filho RM : Synthesis and Characterizations of Poly (Lactic Acid) by Ring-Opening Polymerization for Biomedical Applications. *Chem Eng Trans.* 2014;38:331–336. 10.3303/CET1438056

[11] Xavier MV Macedo MF Benatti ACB : PLLA Synthesis and Nanofibers Production: Viability by Human Mesenchymal Stem Cell from Adipose Tissue. *Procedia CIRP.* 2016;49:213–221. 10.1016/j.procir.2015.11.019

[12] Benatti ACB Xavier MV Macedo MF : Comparative Analysis of Biocompatibility between Poly (L-lactic Acid) (PLLA) and PLDL Purac® Nanofibers for use in Tissue Engineering. *Chem Eng Trans.* 2016;49:199–204. 10.3303/CET1649034

[13] Biswas MC Jony B Nandy PK : Recent Advancement of Biopolymers and Their Potential Biomedical Applications. *J Polym Environ.* 2021. 10.1007/s10924-021-02199-y

[14] Grand View Reseach. Biomaterials Market Size, Share & Trends Analysis Report By Product (Natural, Metallic, Polymer), By Application (Cardiovascular, Orthopedics, Plastic Surgery), By Region, And Segment Forecasts, 2020 – 2027. Reference Source

[15] National Center for Biotechnology Information: "PubChem Substance Record for SID 24890061, SID 24890061, Source: Sigma-Aldrich".PubChem, Accessed 27 July, 2021. Reference Source

[16] National Center for Biotechnology Information: "PubChem Substance Record for SID 24858300, 3,6-Dimethyl-1,4-dioxane-2,5-dione, Source: Sigma-Aldrich".PubChem, Accessed 27 July, 2021. Reference Source

[17] Shahi S Rahimi S Lotfi M : A Comparative Study of the Biocompatibility of Three Root-end Filling Materials in Rat Connective Tissue. *J Endod.* 2006;32(8):776–780. 10.1016/j.joen.2006.01.014 16861081

[18] Filardo G Perdisa F Gelinsky M : Novel alginate biphasic scaffold for osteochondral regeneration: an *in vivo* evaluation in rabbit and sheep models. *J Mater Sci Mater Med.* 2018;29(6):74. 10.1007/s10856-018-6074-0 29804259

[19] Pearce AI Richards RG Milz S : Animal models for implant biomaterial research in bone: a review. *Eur Cell Mater.* 2007;13:1–10. 10.22203/ecm.v013a01 17334975

[20] Henderson CR : Estimation of genetic parameters. *Annals of Mathematical Statistics.* 1950;21:309–310. Reference Source

[21] Akaike H : Information theory and an extension of the maximum likelihood principle. *proceedings of the 2nd international symposium on information*, bn petrow, f. Czaki, Akademiai Kiado, Budapest,1973. Reference Source

[22] Di Rienzo JA Casanoves F Balzarini MG : InfoStat versión 2020.Centro de Transferencia InfoStat, FCA, Universidad Nacional de Córdoba, Argentina. Reference Source

[23] Xavier MV Farez N Salvatierra PL : ARRIVE Questionnaire, CONSORT Check-list and Flow Diagram + TGO, TGP, HEMOGRAMA AND OSTEOREGENERATIVE DATA - Paper: "Biological performance of a bioabsorbable Poly (L-Lactic Acid) produced in polymerization unit: *in vivo* studies".[Data set]. *Zenodo.* 2021. 10.5281/zenodo.5396921 PMC8729025 35035900

[24] Coletta DJ Lozano D Rocha-Oliveira AA : Characterization of Hybrid Bioactive Glass-polyvinyl Alcohol Scaffolds Containing a PTHrP-derived Pentapeptide as Implants for Tissue Engineering Applications. *Open Biomed Eng J.* 2014;8:20–7. 10.2174/1874120701408010020 24772196 PMC3999709

[25] Rentsch C Schneiders W Manthey S : Comprehensive histological evaluation of bone implants. *Biomatter.* 2014;4:e27993. 10.4161/biom.27993 24504113 PMC3979890

[26] Anderson JM Rodriguez A Chang DT : Foreign body reaction to biomaterials. *Semin Immunol.* 2008;20(2):86–100. 10.1016/j.smim.2007.11.004 18162407 PMC2327202

[27] Vert M Mauduit J Li S : Biodegradation of PLA/GA polymers: increasing complexity. *Biomaterials.* 1994;15(15):1209–13. 10.1016/0142-9612(94)90271-2 7703316

[28] Kroeze RJ Helder MN Govaert LE : Biodegradable Polymers in Bone Tissue Engineering. *Materials.* 2009;2(3):833–856. 10.3390/ma2030833

[29] Domingues RCC Pereira CC Borges CP : Morphological control and properties of poly(lactic acid) hollow fibers for biomedical applications. *J Appl Polym Sci.* 2017;134(47):45494. 10.1002/app.45494

[30] Witherel CE Abebayehu D Barker TH : Macrophage and Fibroblast Interactions in Biomaterial-Mediated Fibrosis. *Adv Healthc Mater.* 2019;8(4):e1801451. 10.1002/adhm.201801451 30658015 PMC6415913

[31] Mariani E Lisignoli G Borzì RM : Biomaterials: Foreign Bodies or Tuners for the Immune Response? *Int J Mol Sci.* 2019;20(3):636. 10.3390/ijms20030636 30717232 PMC6386828

[32] Wilson CJ Clegg RE Leavesley DI : Mediation of biomaterial-cell interactions by adsorbed proteins: a review. *Tissue Eng.* 2005;11(1–2):1–18. 10.1089/ten.2005.11.1 15738657

[33] Carnicer-Lombarte A Chen ST Malliaras GG : Foreign Body Reaction to Implanted Biomaterials and Its Impact in Nerve Neuroprosthetics. *Front Bioeng Biotechnol.* 2021;9:622524. 10.3389/fbioe.2021.622524 33937212 PMC8081831

